# People With Dementia Disclosing Their Diagnosis to Social Networks: A Systematic Review and Meta-Synthesis

**DOI:** 10.1093/geront/gnae186

**Published:** 2024-12-18

**Authors:** Gianna Kohl, Mauricio Molinari Ulate, Jem Bhatt, Jennifer Lynch, Katrina Scior, Georgina Charlesworth

**Affiliations:** UCL Unit for Stigma Research, Research Department of Clinical, Educational and Health Psychology, University College London, London, UK; Psycho-Sciences Research Group, Institute of Biomedical Research of Salamanca, University of Salamanca, Salamanca, Spain; UCL Unit for Stigma Research, Research Department of Clinical, Educational and Health Psychology, University College London, London, UK; School of Health and Social Work, University of Hertfordshire, Hatfield, UK; UCL Unit for Stigma Research, Research Department of Clinical, Educational and Health Psychology, University College London, London, UK; UCL Unit for Stigma Research, Research Department of Clinical, Educational and Health Psychology, University College London, London, UK; Research and Development, North East London NHS Foundation Trust, London, UK

**Keywords:** Personal experiences, Qualitative methods, Self-disclosure, Stigma, Well-being

## Abstract

**Background and Objectives:**

Given the stigma of dementia, individuals with the condition may be wary to disclose their diagnosis to other people, both in face-to-face and digital settings. While sharing one’s dementia diagnosis with others is essential for accessing valuable support for social, cognitive, and physical well-being, this area of research has largely been neglected. In this meta-synthesis, we aimed to systematically review qualitative research on the factors associated with online and offline self-disclosure in people with dementia.

**Research Design and Methods:**

We conducted a systematic search in 6 electronic databases. Inclusion criteria comprised qualitative and mixed-methods studies describing experiences with self-disclosure in people with any type of dementia. Quality of the included studies was assessed using the Mixed Methods Appraisal Tool. The meta-synthesis was conducted in NVivo using a thematic synthesis approach.

**Results:**

28 studies were included. 3 analytical themes were generated: “Concealment,” “Stigma and fear,” and “Taking control,” the latter 2 with subthemes. Findings from this review were corroborated with people with dementia and family carers as part of Patient and Public Involvement meetings. Our findings reveal that while stigma plays a pivotal role, people with dementia can take control of the meaning of their diagnosis through self-disclosure.

**Discussion and Implications:**

Self-disclosure is complex and multifaceted. People with dementia, particularly those experiencing stigma, can benefit from post-diagnostic support that encompasses resources and interventions for self-disclosure. Further research is required to investigate people with dementia’s disclosure decision-making process.

## Background and Objectives

Receiving a dementia diagnosis can be an emotional and life-altering experience for individuals (Robinson et al., 2011). One cause of concern may be the stigma associated with dementia, with stigma referring to an “attribute that is deeply discrediting” (Goffman, 1963, p. 63). Dementia-related stigma, stemming from misconceptions and societal attitudes surrounding the condition, has been shaped by historical socio-cultural factors (Nguyen and Li, 2020). In the early stages of the condition, dementia can, to some extent, be concealed from others, making it a concealable stigmatized identity (Quinn and Earnshaw, 2013). Amidst a plethora of decisions faced by people affected by dementia, disclosing the diagnosis becomes a pivotal one, involving considerations of how much to divulge and to whom. Self-disclosure, defined as intentionally communicating or revealing personal information (Chaudoir and Fisher, 2010), offers individuals the opportunity to express personal thoughts, needs and feelings, and build relationships with others (Masur, 2019). Yet, individuals may opt not to disclose personal information, thereby choosing to conceal parts of themselves, due to concerns about potential adverse consequences.

A previous systematic review identified stigmatization and anticipated negative responses as primary factors for concealment in people with mental health problems (Grice et al., 2017). Brohan et al. (2012) found similar reasons in their exploration of self-disclosure of mental health problems in the workplace, alongside positive experiences such as emotional support and work-related adjustments. Self-disclosure is crucial as it positively affects the emotional well-being and social interactions of people with concealable stigmatized identities (Camacho et al., 2020). When individuals choose to disclose their identity, they can foster understanding, reduce feelings of isolation, and access support and resources. Conversely, hiding one’s identity can lead to increased stress and social withdrawal. Unlike people with a noticeable stigmatized attribute, people with a concealable stigmatized identity must navigate self-disclosure decisions almost daily (Chaudoir and Fisher, 2010). Concealing such an identity is associated with social withdrawal (Camacho et al., 2020), heightened social anxiety, as well as decreased quality of life and self-esteem (Hagger & Riley, 2019). Concealment is also prevalent in people with dementia, with a global survey finding that a quarter of individuals reported hiding their diagnosis due to stigma (Alzheimer’s Disease International, 2019). However, while self-disclosure has been extensively studied in various health conditions such as cancer (Henderson et al., 2002) and epilepsy (Pembroke et al., 2017), factors specific to dementia remain insufficiently understood.

Social media serves as a valuable tool for individuals to communicate about their stigmatized identity to provide and seek support (Coulson, 2005). Research also suggests that individuals with concealable stigmatized identities use social media to disclose their identity and share personal illness experiences. For example, Sannon et al. (2019) interviewed 19 people with invisible chronic health conditions and found that participants sought and received informational and emotional support on various platforms (e.g., Facebook, Reddit), growing more comfortable with disclosure over time as they became more experienced with their conditions.

In the dementia field, research has extensively looked at the process of health professionals disclosing the diagnosis to individuals and the impact on them, with literature reviews having explored: the process of sharing the diagnosis *with* patients and family members (Bamford et al., 2004; Werner et al., 2013); ethical and practical issues around health professionals disclosing the diagnosis (Carpenter and Dave, 2004); people with dementia’s preferences regarding receiving the diagnosis (van den Dungen et al., 2014); the impact of receiving the diagnosis on individuals (Mitchell et al., 2013; Robinson et al., 2011); and challenges in sharing and receiving the diagnosis from the perspective of patients, family carers, and healthcare professionals (Yates et al., 2021). However, no literature review has explored disclosure *by* people with dementia to others in a social context. Considering that disclosing one’s diagnosis is essential for accessing post-diagnostic support and care, as well as for remaining socially, cognitively, and physically active, research on self-disclosure in people with dementia is crucial. Through identifying and synthesizing relevant studies, this systematic review aimed to explore the factors involved in the decision to disclose a dementia diagnosis to other people, both online and offline.

## Method

Our systematic review conforms to PRISMA 2020 guidelines (Page et al., 2021) and is registered on PROSPERO (registration number: CRD42020192495). The PRISMA checklist is available in Supplementary File 1 in the Supplementary Material. The protocol and initial search were part of a wider literature appraisal exploring self-disclosure in people with chronic neurological conditions. This systematic review focuses on dementia.

### Search Strategy and Eligibility Criteria

We developed a comprehensive search strategy in collaboration with a subject librarian from University College London and searched six electronic databases (MEDLINE, PsycINFO, Embase, Emcare, CINAHL, and Scopus) in June 2020, updated on January 15, 2024. The updated search focused on dementia and qualitative studies to avoid heterogeneity. Search terms were tailored to each database. Reference lists of included studies were manually screened in July 2020 and on February 4, 2024 to identify additional relevant studies. The search strategy, detailed in Supplementary File 2, had no language or date restrictions.

The eligibility criteria were developed using the Sample, Phenomenon of Interest, Design, Evaluation, and Research (SPIDER) tool (see Table 1), developed for qualitative and mixed-methods syntheses (Cooke et al., 2012). Studies were included if (a) participants were over 18 and had been diagnosed with a form of dementia; (b) they described disclosure experiences in social contexts outside of the workplace; and (c) they were qualitative or mixed-methods research with a qualitative component. Studies were excluded if (a) participants were health professionals disclosing a diagnosis to a patient; and (b) they constituted theses, abstracts, editorials, or non-data papers and were non-peer reviewed. Studies collecting data from dyads were eligible if the findings described self-disclosure by the person with dementia and data were collected simultaneously from both participants.

**Table 1.** Eligibility Criteria for Included Studies Based on the SPIDER Tool

SPIDER	Description
Sample	People with dementia
Phenomenon of interest	Self-disclosure of a dementia diagnosis by the diagnosed individual to other people outside of the workplace
Design	Qualitative or mixed-methods (with a qualitative component) research study
Evaluation	Views, experiences, attitudes, perceptions, beliefs, or feelings regarding self-disclosure
Research type	Peer-reviewed journal articles

*Note*: SPIDER = Sample, Phenomenon of Interest, Design, Evaluation, Research type.

### Study Selection and Data Extraction

Records retrieved from the database searches were exported to EndNote X9 and duplicates removed. Titles and abstracts were screened by one reviewer (GK), with authors of potentially relevant abstracts being contacted for peer-reviewed full-text articles. Two reviewers (GK, MMU) independently assessed full-text articles. Deepl and ChatGPT were used for translations of non-English, German, or Dutch articles, as suggested by Chen (2023). Discrepancies in study eligibility were resolved through discussion with a third reviewer (GC). Data extraction of included studies included author(s), publication year, country, study aim, design, participant characteristics, and key findings using a standardized form developed for this study.

### Quality Appraisal

The methodological quality of included articles was assessed using the Mixed Methods Appraisal Tool (Hong et al., 2018). This tool was chosen for its ability to evaluate various study designs, including qualitative and mixed-methods studies. It comprises two screening questions and five additional questions related to the study design. Outcomes to the questions are reported as “yes”, “no”, or “can’t tell” if information is missing. The appraisal was conducted independently by two reviewers (GK, JB) using Microsoft Excel, with disagreements resolved through discussion. Due to ongoing debates on appraising qualitative studies (Campbell et al., 2011), no overall scoring was applied, and studies were not excluded based on appraisal results.

### Synthesis of Findings

We tabulated, extracted, and synthesized study and participant characteristics narratively. NVivo 12 was used to aid the meta-synthesis, based on guidelines for thematic synthesis (Lachal et al., 2017; Thomas and Harden, 2008). Thematic synthesis was chosen for its ability to systematically integrate qualitative data, allowing for the identification and interpretation of key themes across diverse studies, thus providing a comprehensive understanding of self-disclosure. We coded the results sections of articles line-by-line, capturing both participants’ quotes (first-order constructs) and authors’ interpretations (second-order constructs). If dyads had been interviewed simultaneously, quotes from the family carer were eligible if they built on the person with dementia’s quotes. We created codes inductively, grouping them hierarchically to develop descriptive themes that aligned closely with the original studies. Overarching analytical themes were generated through further interpretation of the descriptive themes in relation to our research aim, “moving beyond” the original studies. A collaborative approach was applied throughout the synthesis. Two reviewers (GK, MMU) independently conducted coding and theme development, which were refined in consultation with two other reviewers. All reviewers approved the final set of themes.

### Patient and Public Involvement (PPI)

We conducted two separate discussions with two different PPI groups, convened by the University of Hertfordshire, to establish if the relevant findings of included studies reflected the experiences of people with dementia. The groups, established by the University of Hertfordshire and a regional research collaboration, included people with dementia and individuals supporting a person with dementia. PPI contributors received an information sheet with examples of relevant findings from included studies prior to the meeting. The discussions took place on Zoom and were facilitated by researchers from the University of Hertfordshire. They were guided by principles for involving PPI contributors in systematic reviews (Brooks et al., 2017), which highlight how such exercises aid in assessing relevance and validity of findings for people with lived experiences. These discussions constituted involvement, not data collection, and as such did not require ethical approval from the university or informed consent (Bunn et al., 2015). No personal or demographic information was collected, and contributors were compensated for their time and expertise by the organizations managing these groups in line with best practices (National Institute for Health and Care Research, 2019).

### Findings

#### Study selection

**Figure 1.**
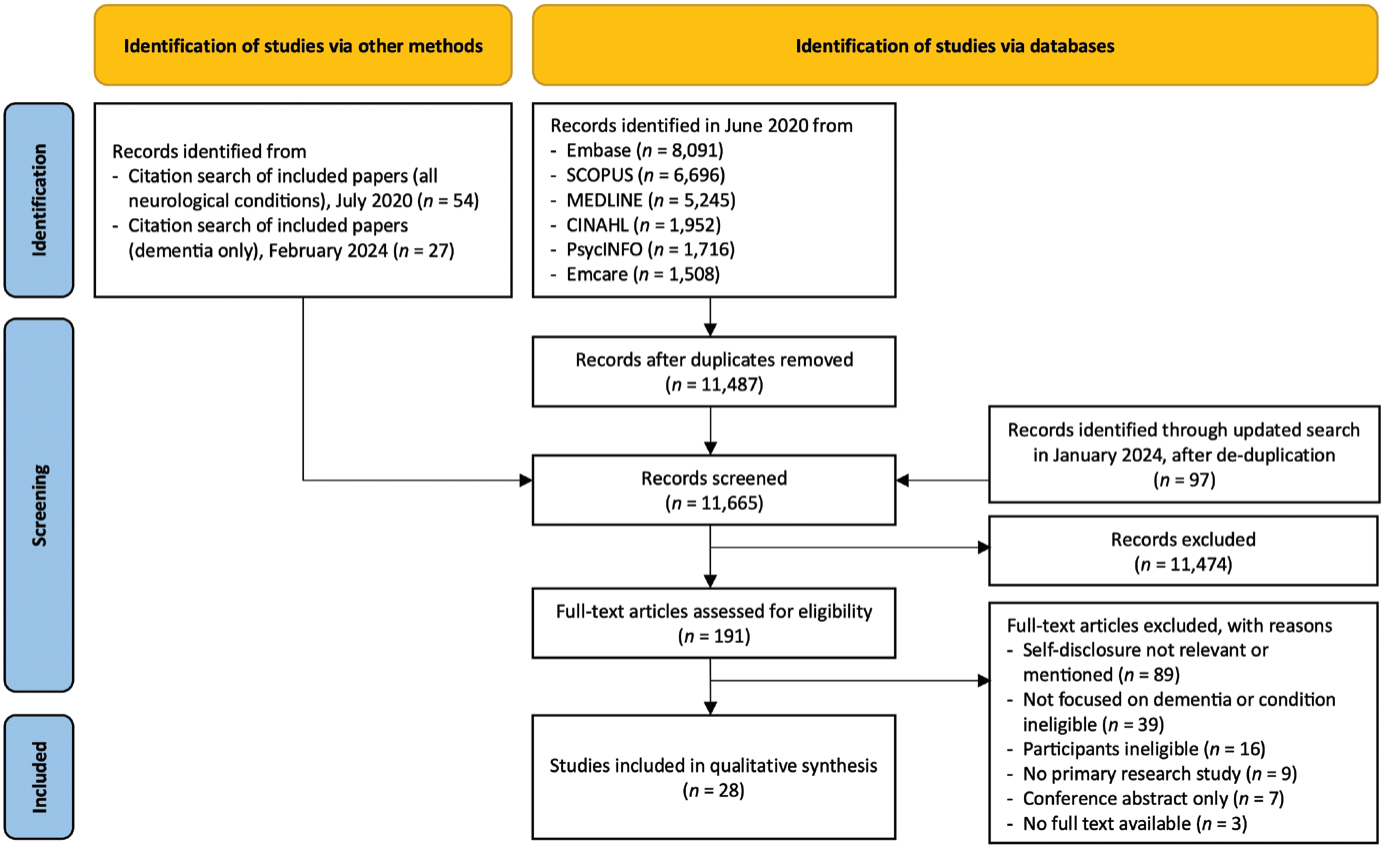
PRISMA flow chart.

The PRISMA flow diagram in Figure 1 details the screening and selection process. The initial search identified 25,208 records, reduced to 11,487 after duplicate removal. An additional 97 records were found in the updated search. The titles and abstracts of 11,665 records were screened. Full-text screening was conducted for 191 articles. This procedure identified 28 relevant studies.

#### Description of included studies

Full characteristics of included studies can be found in Supplementary File 3. Studies were published between 1999 and 2023, with most (*n* = 21) published after 2013. Five studies specifically focused on exploring self-disclosure in people with dementia, and four studies described findings related to disclosure in online spaces. The studies were primarily conducted in high-income countries in Europe (*n* = 17), North America (*n* = 6), and Australia (*n* = 2). Two studies each were conducted in Brazil and Chile. Two studies analyzing publicly available blog data and forum posts did not specify where the creators originated from.

Six studies focused on people with early-stage dementia and three on young-onset dementia (i.e., diagnosed before the age of 65). Nine studies also included family carers as participants. Participant numbers were generally small, with sample sizes ranging between three and 61. In total, the studies represented 321 people with dementia and 84 family carers. Talbot and Coulson (2023) did not specify the number of forum users the analyzed posts originated from. Full demographic characteristics of participants were not always provided. Of the studies that did, ages ranged between 49 and 92, representing the experiences of 160 female and 140 male people with dementia. Five studies reported the ethnic backgrounds of participants (*n* = 37), of which the majority (*n* = 29) identified as White, followed by Black Caribbean (*n* = 3) and African American (*n* = 2).

All studies described general self-disclosure, where the target of self-disclosure was not specified. Additionally, some studies explicitly mentioned the target of disclosure and level of connection: family (*n *= 11), friends (*n* = 9), acquaintances (*n* = 3), other people affected by dementia (*n* = 2), healthcare professionals (*n* = 2), and neighbors (*n* = 1). The setting of self-disclosure was mostly missing. Weaks et al. (2015) described a participant disclosing to a friend during lunch, while another study referred to a social group as the setting for concealment (Telenius et al., 2020).

Studies applied mostly cross-sectional designs, with one study using a longitudinal approach. Twenty-seven studies were qualitative, while one study adopted a mixed-methods approach with a qualitative component. Methods for data collection consisted mainly of interviews (*n* = 24), with a mix of methods being used in three studies. Two studies conducted analysis of publicly available blog and forum data. Four studies collected data online due to COVID-19. Data analysis methods varied, with most studies using thematic analysis (*n* = 10), grounded theory (*n* = 5), or content analysis (*n* = 5).

Detailed results of the quality appraisal can be found in Supplementary File 4. Generally, the studies were well-reported. Inadequate or missing information resulted in incoherence between data sources, collection, analysis, and interpretation in four studies.

#### Thematic meta-synthesis

**Figure 2.**
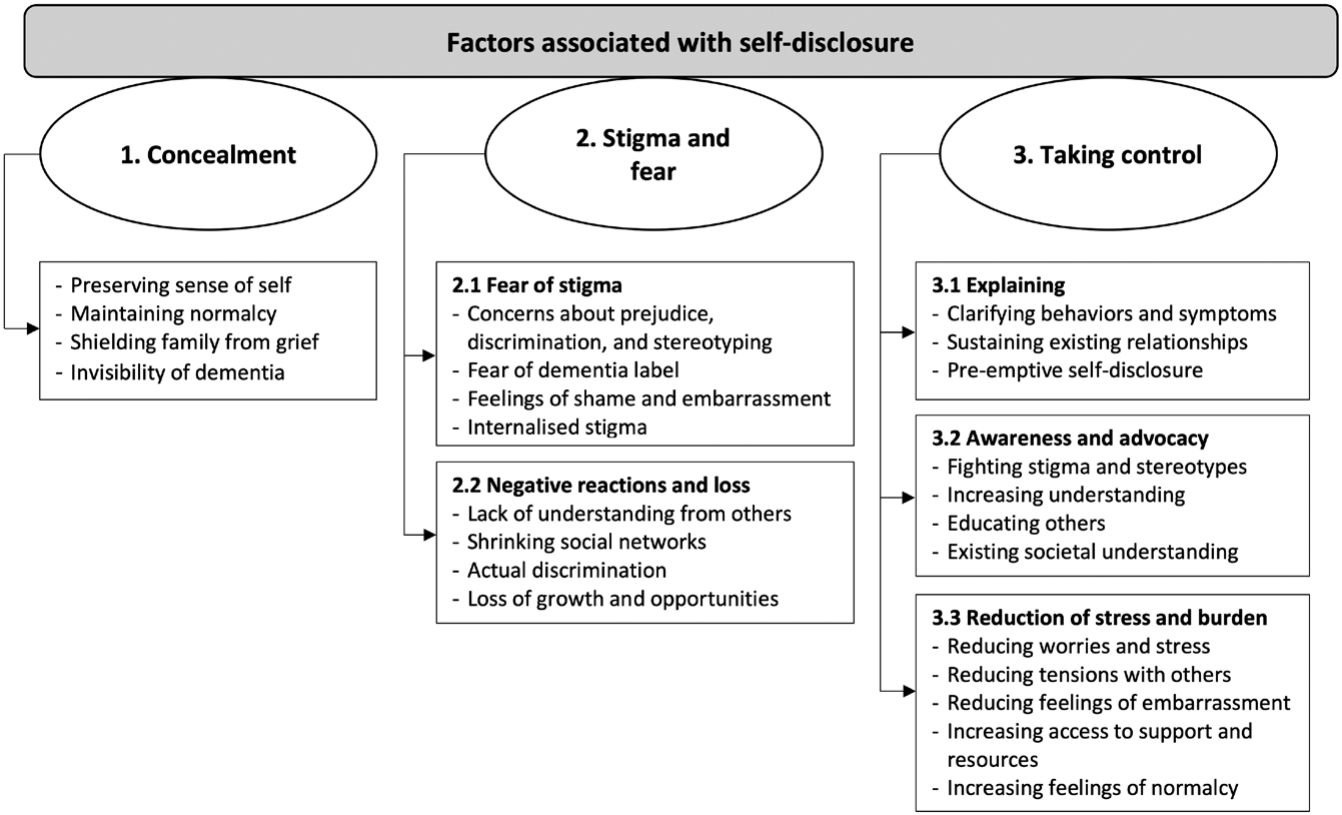
Diagram providing an overview of factors associated with self-disclosure in people with dementia from the thematic meta-synthesis.

The studies described a range of factors contributing to an individual’s decision to disclose their dementia diagnosis to others. These were grouped into three overarching analytical themes called “concealment,” “stigma and fear,” and “taking control”, the latter two themes each contained two and three subthemes, respectively. Figure 2 provides an overview of the analytical themes and subthemes in the form of a diagram, while Table 2 shows which studies are supporting each theme and subtheme. Among the five studies focusing on self-disclosure as a primary research question, there was a notable emphasis on the third theme, “taking control”. These studies consistently featured both first- and second-order constructs related to this theme, whereas “concealment” and “stigma and fear” were represented in two and four of these studies, respectively. Example quotes of these constructs identified for each analytical theme are available in Supplementary File 5.

**Table 2.** Articles Contributing to Generated Themes and Subthemes

Themes	Concealment	Stigma and fear		Taking control		
Study		Fear of stigma	Negative reactions and losses	Explaining	Awareness and advocacy	Reduction of stress and burden
Bielsten et al. (2018)					X	X
Castaño (2020)	X	X			X	
Gajardo et al. (2021)			X			X
Glavind (2023) ^a^			X	X		X
Harris (2012)				X		
Hedman et al. (2013)				X		X
Hellström and Torres, (2013) ^a^	X	X	X	X		
Husband (1999)	X	X				
Husband (2000)	X	X				
Johannessen et al. (2018)	X	X				
Johnson et al. (2022)					X	
Kohl et al. (2023) ^a^					X	X
Langdon et al. (2007)	X	X			X	
Lo et al. (2022)	X	X				
MacRae (2008)		X		X	X	
O’Connor et al. (2018) ^a^		X	X	X	X	X
Oliveira et al. (2023)		X				
Pesonen et al. (2013)	X					
Pipon-Young et al. (2012)	X	X		X		
Stockwell-Smith et al. (2019)	X	X		X		
Talbot and Coulson (2023)	X					X
Telenius et al. (2020)		X				
Thoft and Ward (2022)					X	X
Weaks et al. (2015) ^a^	X	X		X	X	X
Werezak & Stewart (2009)		X	X			
Williamson and Paslawski (2016)				X	X	X
Windle et al. (2023)					X	X
Xanthopoulou and McCabe (2019)	X	X				

*Note*:

^a^Primary research question had focus on self-disclosure.

##### Theme 1: Concealment

The theme “concealment” was apparent in 13 studies illustrating participants’ worries about maintaining secrecy or efforts to actively hide their diagnosis for various reasons. Participants discussed wanting to diminish the effect of the diagnosis on themselves and “not enlarge it by talking about it” (Johannessen et al., 2018, p. 5) or be seen as “normal,” thereby presenting their old or intact selves (Johannessen et al., 2018; Pipon-Young et al., 2012; Weaks et al., 2015; Xanthopoulou and McCabe, 2019). Difficulties accepting the diagnosis were discussed as leading to concealment in one study (Stockwell-Smith et al., 2019). In the study by Langdon et al. (2007), the authors described some participants concealing their diagnosis even when memory problems became apparent to others.

In addition to maintaining normalcy, participants concealed their diagnosis to shield family, friends, and significant others from potential worries and grief about future losses associated with dementia (Husband, 1999; Lo et al., 2022; Pesonen et al., 2013; Talbot and Coulson, 2023; Weaks et al., 2015). This was characterized by phrases suggestive of family and friends spending “many sleepless nights crying” (Pesonen et al., 2013, p. 493).

In several studies, it was suggested that participants used the invisible nature of their condition to hide their diagnosis from others (Castaño, 2020; Hellström and Torres, 2013; Johannessen et al., 2018). This behavior may stem from a desire to mitigate the effect of the diagnosis on others, who may perceive the participant needing to “pull [themselves] together” (Johannessen et al., 2018, p. 5), a lack of understanding from others who “did not quite grasp that something was wrong” (Hellström and Torres, 2013, p. 161), or a belief that “skills are still good enough” (Castaño, 2020, p. 124) to pass as healthy as one blogger described.

##### Theme 2: Stigma and fear

The theme “stigma and fear” was found to be key in 18 studies, including the four studies that focused on self-disclosure in face-to-face contexts. Two subthemes, “fear of stigma” and “negative reactions and losses,” were identified summarizing how stigma and fear influenced self-disclosure.

###### Subtheme 1: Fear of stigma

Sixteen studies described concerns that disclosing the diagnosis would lead to stereotyping, prejudice, or discrimination. Participants worried that others might be judgemental or think less of them, that others would “watch out” for embarrassing things to happen, or that existing relationships would change (O’Connor et al., 2018; Oliveira et al., 2023; Weaks et al., 2015; Werezak and Stewart, 2009; Xanthopoulou & McCabe, 2019). Participants described being worried about the “label of dementia”, with participants associating dementia strongly with “mental illness” or being “crazy” (Hellström and Torres, 2013; O’Connor et al., 2018; Stockwell-Smith et al., 2019; Xanthopoulou and McCabe, 2019). There was a concern that self-disclosure would result in actual negative treatment, including “being taken away” (Stockwell-Smith et al., 2019). Similarly, studies described a sense of shame and embarrassment among participants, including a fear of being called incompetent and being laughed about, and a fear of changes in other people’s attitudes and treatment toward them (Castaño, 2020; Husband, 2000; Langdon et al., 2007; Oliveira et al., 2023). Telenius et al. (2020) described how one participant, who was not open about her diagnosis, had chosen to leave a social group due to criticism from others. As such, fear of judgment had led her to withdraw from an activity she enjoyed.

In some studies, remarks made by participants suggested that they themselves held negative beliefs about the diagnosis (Langdon et al., 2007; Lo et al., 2022; Xanthopoulou and McCabe, 2019). This included saying that dementia “can make you sound as if you’re very gnarled” (Langdon et al., 2007, p. 10) or show that “you are ready for a nursing home” (Xanthopoulou and McCabe, 2019, p. 5).

###### Subtheme 2: Negative reactions and losses

While negative responses or reactions by others upon disclosure were described in several studies, five studies documented how participants became more reluctant to disclose because of this. Friends had started to pay close attention to them or would talk about them behind their back, which affected participants’ self-confidence (Hellström and Torres, 2013; Werezak and Stewart, 2009). In other studies, the opposite was described. According to one participant interviewed by Gajardo et al. (2021), people had started to ignore her once they knew about the diagnosis. Two participants interviewed by Glavind (2023) described feeling either pitied, with friends having lost interest in them, or ignored at social gatherings. Similar experiences were discussed by O’Connor et al. (2018), with participants noting a loss of opportunities and activities due to disclosure. As a result, some had become more selective in whom they would share their diagnosis with, as explained by one person with dementia who wanted to take on a volunteering role:

*I was following the practice to always tell people I had Alzheimer’s. Well the last couple of times, as soon as the Alzheimer’s word came out, the interview [for the volunteering position] cooled. I didn’t get a phone call back so sometimes now I don’t tell people.* (O’Connor et al., 2018, p. 48)

The authors note, however, that several participants who described instances of negative treatment had difficulty naming these experiences as discrimination. O’Connor et al. (2018) suggest that they “were denied the experience of feeling justifiably angry. Instead, [there was a] tendency to discount or brush off one’s feelings …” (p. 50).

##### Theme 3: Taking control

The final theme, identified in 19 studies, describes participants actively disclosing their diagnosis to take control over perceptions associated with dementia. Participants illustrated that openness about their diagnosis increased feelings of empowerment, control, and normalcy. One participant in the study by O’Connor et al (2018) explained that self-disclosure was a way “to empower yourself to take charge of the situation” (p. 49). In their group discussions, the participants noted that one had to be open about the condition in order to manage it “in the best way possible for your own well-being” (p. 49) or to access support. Within this main theme, three subthemes were identified: “explaining”, “awareness and advocacy”, and “reduction of stress and burden”.

###### Subtheme 1: Explaining

In ten studies, participants rationalized that disclosing their diagnosis was a way to explain symptoms and to avoid others guessing the nature of possibly odd or questionable behaviors. This is illustrated in a quote by one participant in the study by Harris (2012) who explained that she had disclosed her condition to friends because “they will wonder how come you don’t remember this?” (p. 311). Disclosure, in turn, was found to be important to sustain existing relationships. In seven studies, self-disclosure was used to make others aware that dementia-related behaviors were out of their control, as suggested by one participant who disclosed so others “could understand. Because I know that it goes a bit wobbly” (Hedman et al., 2013, p. 726). Related to a perception that others might think negatively about people with dementia, one carer interviewed as part of a dyadic study noted: “…we tell most people, because she’s not just funny in the head, sort of thing. It’s a medical condition…” (Stockwell-Smith et al., 2019, p. 632). Similarly, Glavind (2023) described one participant who revealed his diagnosis to anyone to explain the reasons of his behavior. In support of this endeavor, he had publicly shared his diagnosis on Facebook.

Additionally, some participants noted that they disclosed their condition pre-emptively before they had shown any noticeable behavioral symptoms (MacRae 2008; Williamson and Paslawski, 2016). For example, one participant stated she would “tell people ahead of time so if I make a mistake, I don’t feel silly” (Williamson and Paslawski, 2016, p. 8).

###### Subtheme 2: Awareness and advocacy

People with dementia actively disclosed their diagnosis to raise awareness and understanding among other people, as reported in eleven studies. Participants aimed to position dementia as a condition that is common “like a broken arm or broken leg” (MacRae, 2008, p. 404). In several studies, participants expressed a desire to bring “dementia out of the cupboard” (Castaño, 2020, p. 124) by actively educating others about the condition, hoping to destigmatize dementia as a result (Castaño, 2020; Johnson et al., 2022; Kohl et al., 2023; Langdon et al., 2007; MacRae, 2008; O’Connor et al., 2018; Thoft and Ward, 2022; Williamson and Paslawski, 2016; Windle et al., 2023). For example, O’Connor et al. (2018) described how most participants talked about stigma as a “pervasive problem that needed to be named and addressed” (p. 47). Online disclosure was utilized “to change social perceptions of dementia” (Castaño, 2020, p. 124) and to “raise awareness and break down the stigma” (Kohl et al., 2023, p. 4). In two studies (Bielsten et al., 2018; Weaks et al, 2015), participants felt society had already become more understanding and, as a result, had no hesitation to disclose their diagnosis.

###### Subtheme 3: Reduction of stress and burden

Eleven studies described that self-disclosure proved beneficial as it mitigated the stress and burden experienced by some participants due to their initial reluctance to share their diagnosis. Participants felt relieved after self-disclosure, as it “reduced stress due to not having to cover up symptoms” (Bielsten et al., 2018, p. 1725). It was perceived as difficult “trying to keep everything on the surface going” (Weaks et al., 2015, p. 773), which was echoed by Thoft and Ward (2022). Though most participants interviewed by Gajardo et al. (2021) concealed their diagnosis, one participant explained that informing his family had been helpful to reduce tensions. Self-disclosure also led to reduced feelings of embarrassment (Windle et al., 2023), and increased feelings of normalcy: “I will tell it to all that I get in touch with … Then I don’t have to make myself weird … because I could not stand that” (Hedman et al., 2013, p. 726). Similarly, one participant in the study by O’Connor et al. (2018) did not have difficulties disclosing his diagnosis to strangers, instead wearing a badge in public identifying him as living with dementia to enable support from others. Self-disclosure also enabled access to tangible support and resources, including support for family members (Glavind, 2023; Thoft and Ward, 2022; Weaks et al., 2015; Williamson and Paslawski, 2016), which also applied to online spaces (Kohl et al., 2023; Talbot & Coulson, 2023).

### PPI Contributions

Two PPI discussions, attended by five and seven PPI contributors, respectively, were held in February and March 2021. Contributors agreed with our findings, emphasizing the individual and complex nature of self-disclosure. All had direct experience with self-disclosure, though some disclosed selectively. Among those who openly communicated their condition, a prevailing motivation was to explain behavior to help others comprehend the situation. Some contributors reported a shrinking of their social networks as a result of self-disclosure, highlighting the difficulties associated with dementia-related stigma. It was noted, however, that these reactions had not influenced subsequent self-disclosure.

The contributors also brought new insights to the findings. It was suggested that the person with dementia’s age might influence their disclosure decision-making, with older individuals being potentially less likely to disclose as dementia might be one of many illnesses and therefore not noteworthy. Cultural differences, encompassing upbringing and ethnic background, might influence self-disclosure decisions. Furthermore, the contributors suggested it could be worthwhile to explore reactions from others upon being told about a person’s dementia diagnosis, as people who do not have personal experience with dementia might not know how to respond in that moment. Exploring this aspect could offer valuable insights to support people with dementia in feeling more comfortable sharing their diagnosis.

## Discussion

Our meta-synthesis is the first to examine and synthesize evidence related to factors associated with people with dementia disclosing their diagnosis to social networks. It highlights that self-disclosure is best understood as a multifaceted process, with factors often being connected and rarely existing in isolation. While the number of reviews exploring self-disclosure in concealable stigmatized health conditions in adults is limited, several of them echo this complexity (Brohan et al., 2012; Grice et al., 2017; Guo et al., 2020).

### Principal Findings

Only five of the 28 studies identified had self-disclosure of a dementia diagnosis as their primary focus, suggesting that self-disclosure has largely been neglected in dementia research. It can be assumed, however, that this topic is important to people with dementia since it was discussed as an incidental finding in the remaining studies. Additionally, only four studies described aspects of self-disclosure in an online context. While our findings did not suggest differences between offline and online settings, the limited number of studies found highlights an important aspect for future research. This seems particularly relevant given this population’s use of social media (e.g., Kohl et al., 2023), and the common practice of sharing sensitive health information on such platforms (Sannon et al., 2019).

The stigma of dementia emerged as a key factor associated with self-disclosure, aligning with existing literature (Alzheimer’s Disease International, 2019; Herrmann et al., 2018). Stigma concerns became particularly evident as most studies within this theme described anticipated stigma (i.e., thinking disclosure will provoke negative reactions), as opposed to experienced stigma. Relationships between self-disclosure and anticipated stigma has also been discussed in other systematic reviews (Benson et al., 2015; Howells et al., 2021). Additionally, our findings imply that participants themselves can exhibit internalized negative attitudes, referring to dementia using derogatory language such as being “doolally” (Weaks et al., 2015, p. 772). This is also known as self-stigma (Corrigan and Watson, 2002), which has previously been described in people with dementia (Bhatt et al., 2023). This study highlights the pervasive social impact of a dementia diagnosis, which can lead to experiences of stigma and a loss of social and meaningful opportunities for people with dementia (Biggs et al., 2019).

Self-disclosure was perceived as a means of taking control over the diagnosis and its meaning. This emphasis was notable in all studies focused on self-disclosure, where disclosure was seen as a way of asserting control and empowering individuals. Similar to findings in people with brain injury, our review found that self-disclosure served to explain behavioral changes to avoid others guessing about the nature of their difficulties (Riley and Hagger, 2015). It also offered a reduction of stress associated with presenting oneself inauthentically in social settings, adding to the discussions regarding the self and agency in dementia considering its progressive nature (Caddell and Clare, 2010). Moreover, self-disclosure served as a tool for awareness raising, aligning with the concept of advocacy described in the dementia literature, wherein people affected by dementia actively combat stigma by publicly sharing their experiences, both offline (Seetharaman and Chaudhury, 2020) and online (Anderson et al., 2017).

### Strengths and Limitations

A strength of this review and meta-synthesis is its rigorous methodology, which included a systematic and comprehensive literature search, as well as a quality appraisal and synthesis following established methodology. Our review also benefitted from involving people affected by dementia as PPI contributors to validate findings and highlight areas for future research (Pollock et al., 2017).

This review identified a limited number of studies—only five—that focused on self-disclosure in people with dementia. This reveals both a research gap and potential synthesis bias, which could potentially skew perceptions of factors associated with self-disclosure in this population, as well as influence the robustness of our findings and conclusions. In addition, variations in study designs, participant characteristics, and methodologies across the included studies may influence the consistency and generalizability of our findings. To ensure study quality and reduce heterogeneity, only peer-reviewed qualitative articles were included, which means we possibly overlooked relevant information from other sources like quantitative studies. Another potential limitation is the use of a mixed-methods tool to appraise study quality, as it may not be as specific as tools designed solely for qualitative studies. However, as studies were not excluded based on the appraisal, the influence of using this tool on the data synthesis should be minimal. Future reviews are encouraged to consider using the CASP as an alternative tool for quality appraisal, given its evaluation criteria specifically developed for qualitative studies (Critical Appraisal Skills Programme, 2023). Most studies were conducted in Western high-income countries with predominantly White populations, potentially overlooking cultural and ethnic factors related to self-disclosure in people with dementia. Feedback from PPI contributors emphasized the importance of exploring these factors in future studies. Finally, the data reflect a particular subset of people with dementia: Those in the moderate to severe stages of dementia, and those who may be less able to take actions regarding disclosure were likely excluded. Participants also needed to be open to sharing their views and experiences in settings that required personal contact with researchers. Data collected through more anonymous methods might have yielded different findings and, as a result, could have affected our meta-synthesis.

### Implications for Future Research

This review focused on self-disclosure in people with dementia in a social context *outside* the workplace. Workplace settings were excluded due to the absence of prior reviews exploring dementia-related self-disclosure and the distinct dynamics associated with health disclosure in the workplace (Butler and Modaff, 2016), particularly considering the challenges faced by people with dementia in such environments (Ritchie et al., 2015). Considering these challenges, the increasing likelihood of dementia diagnoses among working-age individuals, and the potential financial implications that can come with disclosing one’s dementia diagnosis, future reviews exploring self-disclosure factors are recommended.

Of the 28 included studies, only five focused on self-disclosure in dementia; in the remaining ones, self-disclosure emerged as an incidental finding. This suggests that self-disclosure warrants further exploration, especially considering the implications of disclosing or concealing a concealable stigmatized identity (Chaudoir and Fisher, 2010). These may include disclosure recipients’ reactions, as suggested by the PPI contributors, or longitudinal data to explore changes over time.

While numerous factors influencing self-disclosure were identified, studies did not explore or describe self-disclosure in the context of managing control over medical decision-making, including potential loss of such control, which is particularly important in the context of terminal conditions (Rodríguez-Prat et al., 2016). Additionally, existing research lacks a comprehensive framework or model explaining the differences between those who disclose and those who do not. Future studies are encouraged to quantitatively explore factors guiding the self-disclosure decision. Stigma resistance, suggested to be related to self-disclosure in people with dementia (O’Connor et al., 2018), might be an important factor to consider. Identifying variables like these could offer valuable insights to support people with dementia in navigating their post-diagnostic disclosure process.

## Conclusion

The decision of people with dementia to disclose their dementia diagnosis to social networks is complex and multiple factors are at play. While a limited number of studies have explored self-disclosure in this population, the topic has largely been neglected in dementia research. This includes the role of technology in self-disclosure. The meta-synthesis, as well as discussions with people affected by dementia, suggest that stigma plays a pivotal role in both the decision to disclose and conceal. More rigorous research is required to understand the experience of people with dementia regarding self-disclosure. This could facilitate the development and evaluation of interventions aimed at addressing anticipated and experienced stigma in people with dementia and supporting them in their disclosure decision-making process.

## Supplementary Material

gnae186_suppl_Supplementary_Materials

## Data Availability

Data used for synthesis is available from the included studies. This review was pre-registered on PROSPERO (registration number: CRD42020192495).
