# Supplementary material for: People With Dementia Disclosing Their Diagnosis to Social Networks: A Systematic Review and Meta-Synthesis
Source: Gerontologist. 2024 Dec 18;65(3):gnae186. doi: 10.1093/geront/gnae186 (PMC11833486; doi:10.1093/geront/gnae186)
Supplement: gnae186_suppl_Supplementary_Materials [file gnae186_suppl_supplementary_materials.docx]

Supplementary Material

Supplementary File 1: PRISMA checklist

| **Section and Topic** | **Item #** | **Checklist item** | **Location where item is reported** |
| --- | --- | --- | --- |
| **TITLE** | | |  |
| Title | 1 | Identify the report as a systematic review. | p. 1 |
| **ABSTRACT** | | |  |
| Abstract | 2 | See the PRISMA 2020 for Abstracts checklist. | p. 2 |
| **INTRODUCTION** | | |  |
| Rationale | 3 | Describe the rationale for the review in the context of existing knowledge. | pp. 3-4 |
| Objectives | 4 | Provide an explicit statement of the objective(s) or question(s) the review addresses. | p. 4 |
| **METHODS** | | |  |
| Eligibility criteria | 5 | Specify the inclusion and exclusion criteria for the review and how studies were grouped for the syntheses. | p. 5 |
| Information sources | 6 | Specify all databases, registers, websites, organisations, reference lists and other sources searched or consulted to identify studies. Specify the date when each source was last searched or consulted. | p. 5 |
| Search strategy | 7 | Present the full search strategies for all databases, registers and websites, including any filters and limits used. | Suppl. File 2 |
| Selection process | 8 | Specify the methods used to decide whether a study met the inclusion criteria of the review, including how many reviewers screened each record and each report retrieved, whether they worked independently, and if applicable, details of automation tools used in the process. | p. 5 |
| Data collection process | 9 | Specify the methods used to collect data from reports, including how many reviewers collected data from each report, whether they worked independently, any processes for obtaining or confirming data from study investigators, and if applicable, details of automation tools used in the process. | p. 5 |
| Data items | 10a | List and define all outcomes for which data were sought. Specify whether all results that were compatible with each outcome domain in each study were sought (e.g. for all measures, time points, analyses), and if not, the methods used to decide which results to collect. | p. 5 |
|  | 10b | List and define all other variables for which data were sought (e.g. participant and intervention characteristics, funding sources). Describe any assumptions made about any missing or unclear information. | n/a |
| Study risk of bias assessment | 11 | Specify the methods used to assess risk of bias in the included studies, including details of the tool(s) used, how many reviewers assessed each study and whether they worked independently, and if applicable, details of automation tools used in the process. | pp. 5-6 |
| Effect measures | 12 | Specify for each outcome the effect measure(s) (e.g. risk ratio, mean difference) used in the synthesis or presentation of results. | n/a |
| Synthesis methods | 13a | Describe the processes used to decide which studies were eligible for each synthesis (e.g. tabulating the study intervention characteristics and comparing against the planned groups for each synthesis (item #5)). | p. 6 |
|  | 13b | Describe any methods required to prepare the data for presentation or synthesis, such as handling of missing summary statistics, or data conversions. | p. 6 |
|  | 13c | Describe any methods used to tabulate or visually display results of individual studies and syntheses. | p. 6 |
|  | 13d | Describe any methods used to synthesize results and provide a rationale for the choice(s). If meta-analysis was performed, describe the model(s), method(s) to identify the presence and extent of statistical heterogeneity, and software package(s) used. | p. 6 |
|  | 13e | Describe any methods used to explore possible causes of heterogeneity among study results (e.g. subgroup analysis, meta-regression). | n/a |
|  | 13f | Describe any sensitivity analyses conducted to assess robustness of the synthesized results. | n/a |
| Reporting bias assessment | 14 | Describe any methods used to assess risk of bias due to missing results in a synthesis (arising from reporting biases). | n/a |
| Certainty assessment | 15 | Describe any methods used to assess certainty (or confidence) in the body of evidence for an outcome. | n/a |
| **RESULTS** | | |  |
| Study selection | 16a | Describe the results of the search and selection process, from the number of records identified in the search to the number of studies included in the review, ideally using a flow diagram. | p. 7; Figure 1 |
|  | 16b | Cite studies that might appear to meet the inclusion criteria, but which were excluded, and explain why they were excluded. | n/a |
| Study characteristics | 17 | Cite each included study and present its characteristics. | Suppl. File 3 |
| Risk of bias in studies | 18 | Present assessments of risk of bias for each included study. | p. 8, Suppl. File 4 |
| Results of individual studies | 19 | For all outcomes, present, for each study: (a) summary statistics for each group (where appropriate) and (b) an effect estimate and its precision (e.g. confidence/credible interval), ideally using structured tables or plots. | Suppl. File 3 |
| Results of syntheses | 20a | For each synthesis, briefly summarise the characteristics and risk of bias among contributing studies. | pp. 7-14 |
|  | 20b | Present results of all statistical syntheses conducted. If meta-analysis was done, present for each the summary estimate and its precision (e.g. confidence/credible interval) and measures of statistical heterogeneity. If comparing groups, describe the direction of the effect. | pp. 7-14 |
|  | 20c | Present results of all investigations of possible causes of heterogeneity among study results. | n/a |
|  | 20d | Present results of all sensitivity analyses conducted to assess the robustness of the synthesized results. | n/a |
| Reporting biases | 21 | Present assessments of risk of bias due to missing results (arising from reporting biases) for each synthesis assessed. | n/a |
| Certainty of evidence | 22 | Present assessments of certainty (or confidence) in the body of evidence for each outcome assessed. | n/a |
| **DISCUSSION** | | |  |
| Discussion | 23a | Provide a general interpretation of the results in the context of other evidence. | pp. 14-15 |
|  | 23b | Discuss any limitations of the evidence included in the review. | pp. 15-16 |
|  | 23c | Discuss any limitations of the review processes used. | pp. 15-16 |
|  | 23d | Discuss implications of the results for practice, policy, and future research. | pp. 16-17 |
| **OTHER INFORMATION** | | |  |
| Registration and protocol | 24a | Provide registration information for the review, including register name and registration number, or state that the review was not registered. | p. 4 |
|  | 24b | Indicate where the review protocol can be accessed, or state that a protocol was not prepared. | p. 4 |
|  | 24c | Describe and explain any amendments to information provided at registration or in the protocol. | n/a |
| Support | 25 | Describe sources of financial or non-financial support for the review, and the role of the funders or sponsors in the review. | p. 18 |
| Competing interests | 26 | Declare any competing interests of review authors. | p. 18 |
| Availability of data, code and other materials | 27 | Report which of the following are publicly available and where they can be found: template data collection forms; data extracted from included studies; data used for all analyses; analytic code; any other materials used in the review. | p. 18 |

*From:*  Page MJ, McKenzie JE, Bossuyt PM, Boutron I, Hoffmann TC, Mulrow CD, et al. The PRISMA 2020 statement: an updated guideline for reporting systematic reviews. BMJ 2021;372:n71. doi: 10.1136/bmj.n71

Supplementary File 2: Example of updated search strategy

| **APA PsycInfo** |
| --- |
| 1 exp dementia/ 93652  2 exp alzheimer's disease/ 56663  3 exp picks disease/ 283  4 exp creutzfeldt jakob syndrome/ 791  5 exp huntingtons disease/ 3611  6 (dement* or Alzheimer* or "cognitive disorder*" or "Pick's disease*" or "vascular dementia" or "Lewy bod* disease*" or "dementia with Lewy bod*" or "Creutzfeldt-Ja#ob" or "mixed dement*" or "frontotemporal dement*").mp. [mp=title, abstract, heading word, table of contents, key concepts, original title, tests & measures, mesh word] 133832  7 Huntington*.mp. [mp=title, abstract, heading word, table of contents, key concepts, original title, tests & measures, mesh word] 5656  8 exp self-disclosure/ 8701  9 exp privacy/ 3781  10 exp Secrecy/ 736  11 exp "Sharing (Social Behavior)"/ 2352  12 (disclos* or self-disclos* or "coming out" or conceal* or self-conceal* or secrecy).mp. [mp=title, abstract, heading word, table of contents, key concepts, original title, tests & measures, mesh word] 51535  13 (diagnosis adj3 (disclos* or conceal* or shar* or communicat* or tell* or talk* or notif* or inform*)).mp. [mp=title, abstract, heading word, table of contents, key concepts, original title, tests & measures, mesh word] 2972  14 8 or 9 or 10 or 11 or 12 or 13 58918  15 1 or 2 or 3 or 4 or 5 or 6 or 7 137916  16 14 and 15 1071  17 limit 16 to up=20200622-20240116 174 |

Supplementary File 3: Characteristics of studies included in review (presented alphabetically).

| Study details | Research aims | Sample | Methodology | Key findings relating to self-disclosure | Disclosure recipient and setting |
| --- | --- | --- | --- | --- | --- |
| 1. Bielsten et al. (2018), Sweden | To identify content for a self-management guide, using outcomes of previous research and data from dyadic interviews | Five married dyads  Two female and three male people with dementia, aged 71-77  All diagnosed with AD | In-person semi-structured interviews and written comments from participants  Thematic analysis, combining inductive and deductive processes | Reasons for disclosure included fighting stigma and reducing concealment-related stress  Disclosure facilitated stress reduction and increased levels of support and understanding | Self-disclosure in general  Setting not provided |
| 2. Castaño (2020), online | To explore how metaphors shape the lived experiences of people with YOD | Internet blogs by ten people with dementia (five female, five male) | Primary content analysis of 622 blog posts publicly accessible on the internet  Metaphor identification procedure and discourse analysis | Reason for disclosure included challenging stereotypes  Having an invisible illness facilitated concealment  Individuals were concerned about potential negative reactions | Self-disclosure in general  Setting not provided |
| 3. Gajardo et al. (2021), Chile | To describe the experiences of living with dementia following diagnosis | Eleven people with dementia (five female, six male), aged 64-82  All diagnosed with AD | In-person interviews  Content analysis using open coding | Reason for concealment included a lack of understanding in others  Disclosure facilitated stress reduction and support  Participants carefully chose who to disclose to, based on a level of trust | Self-disclosure in general and to family  Setting not provided |
| 4. **Glavind (2023), Denmark** | To describe the social implications of living with an AD diagnosis | 13 people with dementia  6 family members  All diagnosed with AD | 12-month ethnographic study over a course of 2.5 years  Fieldwork in three phases, consisting of interviews and observations at participants’ homes | Reasons for disclosure included explaining symptoms and accessing support  Reasons for concealment included actual losses and negative reactions from others and to be seen as normal | Self-disclosure in general and to immediate family, friends, and neighbours  Setting not provided |
| 5. Harris (2012), USA | To explore if remaining friendships have a meaningful impact on the experience of early-stage dementia | Eight people with dementia (all female), aged 59-85  87% AD, 13% other  All identified as White | In-person in-depth interviews  Grounded theory | Reasons for disclosure included explaining symptoms  Not disclosing the diagnosis to friends meant friendships were more difficult to maintain | Self-disclosure to friends  Setting not provided |
| 6. Hedman et al. (2013), Sweden | To describe how people with mild and moderate AD express their sense of self | Twelve people with dementia (five female, seven male), aged 60-80  All diagnosed with AD | In-person semi-structured interviews  Content analysis using established social-constructionist theory of selfhood | Reasons for disclosure included wanting to be oneself and to explain symptoms | Self-disclosure in general  Setting not provided |
| 1. 7. **Hellström & Torres (2013), Sweden** | To explore what couples living with dementia want to know and disclose about the diagnosis | Twenty married dyads  Ten female and ten male people with dementia, aged 61-80  AD was the most common diagnosis | In-person semi-structured interviews  Qualitative analysis identifying themes | Findings presented as themes  Five disclosure patterns: 1) want to know and tell (no reservations about it); 2) want to know and tell (some reservations about it); 3) want to know but do not want to tell; 4) want to know but cannot decide if we want to tell; and 5) cannot agree on neither knowing nor telling  Majority of dyads disclosed openly  Reasons for concealment included uncertainty how to disclose, past negative reactions and treatment, and wishes to not be treated differently  Invisible symptoms impede disclosure  Disclosure was helpful to avoid others guessing | Self-disclosure in general  Setting not provided |
| 1. 8. Husband (1999), UK | To describe the psychological impact of receiving a dementia diagnosis and issues arising from psychotherapeutic work | 3 people with dementia (2 female, 1 male), aged 64-71  All diagnosed with AD | Psychotherapy based on CBT  Descriptions of cases | Reasons for concealment included shame, fear of being laughed at, and sparing loved ones from grief  Concealment led to reduced social activities | Self-disclosure in general and to family, friends, and health professionals  Setting not provided |
| 9. Husband (2000), UK | To explore the proportion of people with dementia capable of engaging in discussion about their diagnosis, worries and effects of these worries | Ten people with dementia (seven female, three male), aged 61-72 | In-person structured interview  Content analysis and frequency counts | Reasons for concealment included being ashamed and fear of negative treatment, including being called incompetent and not being listened to  All participants were worried about maintaining secrecy and others finding out about the diagnosis | Self-disclosure in general  Setting not provided |
| 10. Johannessen et al. (2018), Norway | To explore the existential experiences and coping mechanisms of people with YOD | Ten people with young-onset dementia (seven female, three male), aged 49-67 | Longitudinal exploratory descriptive study with a duration of two years  In-person semi-structured interviews  Modified grounded theory | Reasons for concealment included wishes for normalcy and diminishing the impact of the condition on oneself  Participants controlled their outer image by concealing  Being younger and healthy looking made concealment easier | Self-disclosure in general  Setting not provided |
| 11. Johnson et al. (2022), USA | To explore how people with dementia and family carers engage with online spaces for social support | Four people with dementia (one female, three male), aged 50-72  Two people with dementia identified as Black/African American and two as White  16 family carers | Semi-structured interviews conducted on Zoom  Thematic analysis | Reasons for disclosure included educating others and peer support  Reasons for concealment included shame and stigma  Online spaces provided room for mutual support and awareness raising | Self-disclosure in general  Setting not provided |
| 12. **Kohl et al. (2023), UK** | To describe how people with dementia use social media and share their diagnosis or dementia-related information on social media | Interview sample: 10 people with dementia (seven female, three male), aged 57-80  50% AD, 20% FTD, 10% mixed, 10% PCA, 10% VD | Mixed-methods study, with qualitative component consisting of semi-structured interviews conducted on Zoom  Qualitative content analysis | Reasons for disclosure on social media included raising awareness and understanding, and facilitating support | Self-disclosure in general and peers (other people with dementia)  Specific setting not provided, but disclosure online is described |
| 13. Langdon et al. (2007), UK | To explore how people with dementia‘s perception of their condition and their understanding of others’ reactions to them had changed | Twelve people with dementia (six female, six male) aged 66-87  50% VAD, 33.3% AD, 8.3% FTD, 8.3% LBD  Nine identified as White and three as Black Caribbean | In-person semi-structured interviews  Interpretative phenomenological analysis | Reasons for disclosure included advocacy and educating others  Reasons for concealment included fear of misconceptions, stigma, and being talked about | Self-disclosure in general and to family, friends, and health professionals  Setting not provided |
| 14. Lo et al. (2022), Australia | To examine the affective, behavioural, and cognitive experiences of people with PPA | Six people with dementia (two female, four male), aged 64-79,  Time since diagnosis was 6 months to 2 years  All diagnosed with PPA | Semi-structured interviews conducted on Zoom  Inductive thematic analysis | Reasons for concealment included fearing loss of social relationships and not wanting to burden family and friends  All concealed their diagnosis in the beginning, with some also thereafter  Participants expressed stereotypical notions attached to ‘dementia’ | Self-disclosure in general and to family, (close) friends, and acquaintances  Setting not provided |
| 15. MacRae (2008), Canada | To examine the meaning of AD to people with early-stage AD | Eight people with dementia (two female, six male), aged 60-85  All diagnosed with AD | In-person semi-structured interviews  Themes established through inductive and deductive data analysis | Reasons for disclosure included explaining symptoms and educating people about misconceptions  Reasons for concealment included perceived negative assumptions | Self-disclosure in general  Setting not provided |
| 16. **O’Connor et al. (2018), Canada** | To explore the diagnostic disclosure process for people with dementia in relation to stigma, discrimination, and social citizenship | Eight people with dementia (two female, six male), aged 57-82 | In-person group discussions with monthly meetings over sixteen months  Deductive approach with social citizenship in mind and discourse analysis strategies | Findings presented descriptively  Reasons for disclosure included fighting stigma and educating others, empowering oneself and others, explaining symptoms, and gaining social support  Reasons for concealment included fear that relationships would change, fear of discrimination, and fear of being treated differently  Disclosure could lead to loss of 1) growth opportunities, 2) active participation, and 3) meaningful activities | Self-disclosure in general and to family and friends  Setting not provided |
| 17. Oliveira et al. (2023), Brazil | To explore experiences of self-stigma in people with dementia living in Brazil | Six people with dementia (five female, one male), aged 73-87  All identified as White | In-person semi-structured interviews  Critical Narrative Inquiry  Inductive and deductive analysis, informed by existing stigma theory | Reasons for concealment included fear of judgment and anticipated negative reactions | Self-disclosure in general  Setting not provided |
| 18. Pesonen et al. (2013), Finland | To explore shared experiences of receiving a diagnosis of dementia for people with dementia and family members, and to understand how they manage their lives after diagnosis | Eight dyads  Five female and three male people with dementia, aged 51-74  75% AD, 25% LBD | In-person low-structured, in-depth interviews  Constant comparative analysis using grounded theory | Reasons for concealment included sparing close relatives from grief  people with dementia wanted to remain in control over who would be told about the diagnosis | Self-disclosure in general and to family  Setting not provided |
| 19. Pipon-Young et al. (2012), UK | To explore the broader experiences of people with YOD, including beneficial support as well as areas in need of change | Eight people with young-onset dementia (seven female, one male), aged 60-67  87.5% AD, 12.5% mixed dementia  Six identified as White British and one each as White/Asian and Black | In-person semi-structured interviews  Thematic analysis | Reasons for disclosure included explaining symptoms  Reasons for concealment included uncertainty about possible reactions and perceptions of participants | Self-disclosure in general  Setting not provided |
| 20. Stockwell-Smith et al. (2019), Australia | To explore the impact of early‐stage dementia on dyads’ confidence in their capacity to manage dementia-related changes, and to access appropriate support | Thirteen dyads  Five female and eight male people with dementia, aged 66-87 | In-person semi-structured interviews  Thematic analysis | Reasons for concealment included fear of stigma and difficulties accepting the diagnosis | Self-disclosure in general and to family and friends  Setting not provided |
| 21. Talbot & Coulson (2023), online | To explore the content of messages posted by people with dementia in an online discussion forum | No sample description as forum is anonymous | Analysis of 863 messages posted in 100 randomly selected conversation threads  Reflexive thematic analysis | Reason for disclosure included supporting other people with dementia  Reason for concealment included shielding others from grief | Self-disclosure in general and to family and peers (other people with dementia)  Setting not provided |
| 22. Telenius et al. (2020), Norway | To explore the perceived needs of people with dementia | 35 people with dementia (19 female, 16 men), aged 59-92 | In-person semi-structured interviews  Qualitative content analysis | Reasons for concealment included a perceived lack of understanding  Concealment led to social withdrawal and a decrease in meaningful activities | Self-disclosure in general and to acquaintances  Social group described a setting for self-disclosure |
| 23. Thoft & Ward (2022), Denmark | To explore the lifeworld perspective of people with dementia experiencing and managing everyday life | Twelve people with dementia (three female, nine male), aged 65-82  50% AD, 33.3% unknown, 16.7% VD | In-person semi-structured interviews  Analysis with existing hermeneutic phenomenology in mind | Disclosure helped to alleviate the stress of trying to hide dementia, facilitated support, and help to raise awareness  All participants had self-disclosed | Self-disclosure in general  Setting not provided |
| 24. **Weaks et al. (2015), UK** | To explore the experiences of people with dementia and family members regarding sharing the diagnosis with others | Five people with dementia (three female, two male), aged 68-79  All diagnosed with AD  One family member | In-person interviews and participant observation over a six-month period  Grounded theory | Findings presented descriptively, covering a range of topics related to disclosure, including attitudes of change regarding disclosure over the research period  Reasons for disclosure included explaining symptoms, worries that AD is hereditary, stress of not telling, unburdening carers, feeling that society is more understanding, and to make needs known  Reasons for concealment included privacy, wishes for normalcy, protecting others, embarrassment, fear of stigma, worries of straining the relationship, worries of negative reactions, and others potentially putting information forward  Concealment was associated with social isolation, difficulty accessing appropriate support, and stress | Self-disclosure in general and to family, friends, and acquaintances  Example of self-disclosure at lunch with friends |
| 25. Werezak & Stewart (2009), Canada | To explore the process of learning to live with early-stage dementia | Six people with dementia, aged 61-79  83.3% AD, 16.7% VAD | In-person semi-structured interviews  Grounded theory | Reasons for concealment included uncertainty about potential reactions | Self-disclosure in general and to family and friends  Setting not provided |
| 26. Williamson & Paslawski (2016), Canada | To explore the concept of resilience and factors associated with it among people with dementia and their care partners | Seven people with dementia (four female, three male), aged 65-82  Five family carers | In-person semi-structured interviews  Thematic analysis | Reasons for disclosure included decreasing embarrassment, educating people, explaining symptoms, and facilitating support  Disclosure contributed positively to well-being | Self-disclosure in general  Setting not provided |
| 27. Windle et al. (2023), UK | To develop a conceptual model of resilience in PEOPLE WITH DEMENTIA to inform service development and health and care practices | Eight people with dementia (four female, four male), aged 51-81  37.5% AD, 25% PCA, 25% mixed, 12.5% PSP  Ten family carers | In-person stakeholder engagement meetings and online semi-structured interviews  Thematic analysis | Disclosure led to less personal embarrassment and more public awareness and understanding  Openness about diagnosis acted as resilience reserve | Self-disclosure in general  Setting not provided |
| 28. Xanthopoulou & McCabe (2019), UK | To explore people’s experiences of cognitive decline and receiving a diagnosis of dementia | Sixty-one people with dementia (34 female, 27 male), aged 65-91  60% AD, 20% VAD, 21.2% mixed dementia/other | In-person semi-structured interviews  Thematic analysis | Reasons for concealment included worry of being called incompetent, stigma, and worry of being treated differently  Participants put increasing effort into hiding symptoms | Self-disclosure in general and to family  Setting not provided |

*Note*. AD = Alzheimer’s disease; CBT = Cognitive-behavioural therapy; FTD = Frontotemporal dementia; LBD = Lewy Body dementia; PCA = Posterior cortical atrophy; PPA = Primary progressive aphasia; PSP = Progressive supranuclear palsy; VAD = Vascular dementia. Bold studies indicate those whose primary research question focused on self-disclosure.

Supplementary File 4: Quality appraisal of included studies using the MMAT

|  | Methodological quality of included studies | | | | |
| --- | --- | --- | --- | --- | --- |
| Qualitative study | Is approach appropriate to answer study’s research question? | Are data collection methods adequate? | Are findings adequately derived from data? | Is interpretation substantiated by data? | Is there coherence between data sources, collection, analysis & interpretation? |
| Bielsten et al. (2018) | Y | Y | Y | Y | Y |
| Castaño (2020) | Y | Y | Y | Y | Y |
| Gajardo et al. (2021) | Y | Y | Y | Y | Y |
| **Glavind (2023)** | Y | Y | Y | Y | Y |
| Harris (2012) | Y | Y | N | Y | N |
| Hedman et al. (2013) | Y | ? | Y | Y | ? |
| **Hellström & Torres (2013)** | Y | Y | Y | Y | Y |
| Husband (1999) | Y | Y | ? | N | N |
| Husband (2000) | Y | ? | N | N | N |
| Johannessen et al. (2018) | Y | Y | Y | Y | Y |
| Johnson et al. (2022) | Y | Y | Y | Y | Y |
| **Kohl et al. (2023)** | Y | Y | Y | Y | Y |
| Langdon et al. (2007) | Y | Y | Y | Y | Y |
| Lo et al. (2022) | Y | Y | Y | Y | Y |
| MacRae (2008) | Y | Y | Y | Y | Y |
| **O’Connor et al. (2018)** | Y | Y | Y | Y | Y |
| Oliveira et al. (2023) | Y | Y | Y | Y | Y |
| Pesonen et al. (2013) | Y | Y | Y | Y | Y |
| Pipon-Young et al. (2012) | Y | Y | Y | Y | Y |
| Stockwell-Smith et al. (2019) | Y | Y | Y | Y | Y |
| Talbot & Coulson (2023) | Y | Y | Y | Y | Y |
| Telenius et al. (2020) | Y | Y | Y | Y | Y |
| Thoft & Ward (2022) | Y | Y | Y | Y | Y |
| **Weaks et al. (2015)** | Y | Y | Y | Y | Y |
| Werezak & Stewart (2009) | Y | Y | Y | Y | Y |
| Williamson & Paslawski (2016) | Y | Y | Y | Y | Y |
| Windle et al. (2023) | Y | Y | Y | Y | Y |
| Xanthopoulou & McCabe (2019) | Y | Y | Y | Y | Y |
| Mixed-methods study | Is there an adequate rationale for using a mixed-methods design? | Are the components of the study effectively integrated? | Are the outputs of the integration adequately interpreted? | Are inconsistencies between components’ results adequately addressed? | Do study components adhere to quality criteria of each method used? |
| Kohl et al. (2023) | Y | Y | Y | Y | Y |

*Note*. Y = Yes; N = No; ? = Can’t tell. Bold studies indicate those whose primary research question focused on self-disclosure.

Supplementary File 5: Themes and subthemes with examples of first- and second-order constructs

| **Themes and subthemes** | **Data and studies** | |
| --- | --- | --- |
|  | **First order-constructs** | **Second-order constructs** |
| Concealment | **“The difficult thing is that no one notices anything … people don’t notice that something is wrong.” (Hellström & Torres, 2013, p. 161)**  “I want to live my life as well as possible, and not talk very much about it. Yes, not enlarge it by talking.” (Johannessen et al., 2018, p. 5)  “I think I’m just hiding it. And they [Joshua’s sister] know there’s something wrong, and they know that I wouldn’t miss events unless there was a reason…they have their own problems…I don’t want to load this on her.” (Lo et al., 2022, p. 2487)  “In our rehabilitation course, we talked that we want to spare our close ones, and therefore, we keep the information ourselves. I want to tell when the time is right. Think if I’ll tell my sister, she would spend many sleepless nights crying.” (Pesonen et al., 2013, p. 493)  “I haven’t said anything to anybody … I wouldn’t because they see me as I was before.” (Pipon-Young et al., 2013, p. 606)  “I didn't like to accept it myself, and until I accepted it, I really couldn't pass it on to anybody else.” (Stockwell-Smith, 2019, p. 632)  **“I would prefer to think I am still a bit normal … no doubt it will worsen so I feel that I just should be quiet and wait until it becomes obvious.” (Weaks et al., 2015, p. 772)**  “I have to cover up, you know. Well, say I’m in a conversation with somebody and I have to admit to them either that I have lost the plot completely or some- thing disturbed me or something. I have to find an excuse for not getting it right.” (Xanthopoulou & McCabe, 2019, p. 5) | “Since dementia is not an immediately perceptible condition, at least in the first stages, for some participants deciding not to disclose their diagnosis became a strategy of self-preservation … against the stigmatization and denial that many find when people either do not believe they have dementia or minimize the problem.” (Castaño, 2020, p. 124)  “She found it difficult to talk to friends and relatives because they became upset and she wanted to ‘protect’ them from the implications of her diagnosis.” (Husband, 1999, p. 181)  “They were concerned with maintaining secrecy and often lived in fear of other people finding out.” (Husband, 2000, p. 546)  “All informants, except one, mentioned that they have controlled their outer image by concealing the diagnosis; they have presented their difficulties as a ‘memory problem’. Their reasons are to be seen as ‘normal’, to be able to relate to others as usual, and diminish the impact of the disease … Being and looking rather young, they have misled others into not believing in a diagnosis of dementia.” (Johannessen et al., 2018, p. 5)  “Sometimes, even in social situations when the memory problem became apparent to others, participants chose not to reveal their diagnosis and used other strategies to cope instead.” (Langdon et al., 2007, p. 995) |
| Stigma and fear |  |  |
| Fear of stigma | “When you say the word dementia, people shrink back in horror and then they deny the possibility.” (Castaño, 2020, p. 124)  **“You don’t want to put a label on yourself, you see.” (Hellström & Torres, 2013, p. 162)**  “It is very taboo for me. For others, also—I feel. If you have got Alzheimer, you are ready for a nursing home. I am not going to tell others.” (Johannessen et al., 2018, p. 5)  “Veronica said we must tell them at the club … that I have dementia. But I won’t tell them anything […] I think they may yap, yap, yap with everybody that I have dementia.” (Langdon et al., 2007, p. 995)  **“I think that many of us in the past or present are concerned more about how their relationship will change if that happens.” (O’Connor et al., 2018, p. 47)**  “Ah it gets a bit … (long pause) I think people would distance themselves, wouldn’t they? …We can see [reactions] in certain people, can’t we? … I think people get a bit like … People keep kind of looking [at you], don’t they?” (Oliveira et al., 2023, p. 456)  “I don't expect anything from my neighbours or anything like that. I don't know how many know I have short-term memory loss, or not, but I wouldn't tell them. I think it's an embarrassment’.” (Stockwell-Smith et al., 2019, p. 632)  “I have early onset Alzheimer’s and was diagnosed 8 weeks ago. At ﬁrst, after the initial shock, I seemed to take it in my stride. The hardest thing was telling my husband. We have not told our adult children … My husband is in the angry stage and I’m afraid and the DIY’s getting the brunt of his frustration. :(“ (Talbot & Coulson, 2023), pp. 3-4)  **“I wouldn’t want people to think I was doolally ... I think that could be disastrous.” (Weaks et al., 2015, p. 772)**  “I haven’t really told anyone else, because I ﬁgure if they know they’re always watching for you to do things that are not what you should be doing. [chuckles]” (Werezak & Stewart, 2009, p. 375)  “You don’t know how people will be. I haven’t told my daughter yet. I don’t know whether to or not you see because they start to treat you differently don’t they?” (Xanthopoulou & McCabe, 2019, p. 5) | **“… these people seemed to be aware that disclosure entails talking about a disease to which much stigma is attached; a disease that few have enough information about and understand.” (Hellström & Torres, 2013, p. 163)**  “[She] was highly anxious and described herself as ‘terrified’ by the diagnosis, expressing fears about ‘going mad’ and being ‘put away’. She had told no one but her sister about the diagnosis. She was worried that her neighbours would find out she had AD and was consequently avoiding social activity. She feared rapid deterioration and that she might behave in a socially embarrassing way.” (Husband, 1999, p. 180)  “They believed that others would treat them differently as though they were in a ‘second childhood’ or ‘an idiot’. They were also concerned that they would become incompetent or ‘stupid’, suggesting the potential for negative beliefs about the self.” (Husband, 2000, p. 546)  “The disinclination of some participants to let people in their wider social circles know about their diagnosis may represent resistance to others making assumptions, giving them labels or stereotyping them. Participants were concerned they would be considered ‘funny’, ‘sick’, ‘crackers’ or ‘demented’ if they revealed their diagnosis.” (Langdon et al., 2007, p. 995)  “Participants expressed reticence to reveal their diagnosis in the early days of their diagnosis (and thereafter), fearing loss of friendships and respect.” (Lo et al., 2022, p. 2487)  “Henry was ‘cautious’ about whom he told, believing that AD was a topic that was ‘just getting out of the taboo stage’.” (MacRae, 2008, p. 404)  “When relating to others, people living with dementia expressed active efforts to avoid negative reactions such as anger or judgements directed at them, like being undermined or seen as unreliable or repetitive, which could lead to feelings of shame or embarrassment. In some cases, participants demonstrated a tendency to select to whom they would disclose their diagnosis based on anticipated negative reactions that could come from this.” (Oliveira et al., 2023, p. 455)  “Many participants described ways in which they covered up their dementia. Reasons for this surrounded the uncertainty of others’ reactions and perceptions of them.” (Pipon-Young et al., 2013, p. 606)  “Perceived stigma is also evident in the concern expressed by one care recipient regarding the negative impact disclosure may have on her status within the small retirement community where she and her husband lived. This resulted in her refusal to share her diagnosis or seek support from family or friends.” (Stockwell-Smith et al., 2019, p. 634)  “The impacts of dementia are often not just conﬁned to the individual with the diagnosis, but also have powerful eﬀects on loved ones. Consistent with this, users explained the challenges of disclosing their diagnosis to others, with some users delaying disclosure to certain family members.” (Talbot & Coulson, 2023, p. 3)  “Julie, who had chosen not to be open about her dementia diagnosis, experienced criticism while playing bridge and decided to withdraw from this activity. She explained that she was hurt by the comments from a fellow bridge player but regretted not to be part of that social group anymore.” (Telenius et al., 2020, p. 6)  “… participants unanimously recalled feeling anxious about how others would respond if and when they discovered that the participant had memory loss. Two primary factors they considered when contemplating whether to disclose their memory loss were how people might react if they knew about the dementia and how people had reacted when they discovered the participant had memory loss.” (Werezak & Stewart, 2009, p. 375) |
| Negative reactions and losses | I find that it's not something to go around publicizing because it isolates you () You can be in a group, and they don't pay attention to you because [other people] think you don't know what you're talking about. (Gajardo et al., 2021, p. 189; translated from Spanish)  **“In some ways, I regret telling about my diagnosis…My friends and family pity me instead of seeing me as I am. My friends don’t have the same interest in me as before. I am no longer interesting.” (Glavind, 2023, p. 7)**  **“My broker–I've been dealing with her for 25 years and she doesn't call me anymore. She calls my wife. For 22 years, she never even talked to my wife once. My wife answered the phone and she always asked for me, even on her investments. As soon as my wife told her–I'm the one who told her, I guess. And the next thing you know, she doesn't–and she doesn't do it on purpose. But she just–that's just the way it is.” (O’Connor et al., 2018, p. 48)** | **“She talked about noticing that her friends had begun to observe her more closely once she had disclosed that she had been diagnosed. This affected her self-confidence and was one of the reasons she no longer wanted to talk about her disease with people outside of her immediate family.” (Hellström & Torres, 2013, p. 163)**  “… when the ﬁrst author asked one participant how she knew that members of her women’s group were ‘gossiping’ about her memory loss, she replied, ‘Well, because they’ll go and talk to themselves … and then look at me.’ As a result of this experience, the participant and her husband decided not to tell anyone about her memory loss when they moved to a large city. She said, ‘I just cover it up and no one knows’.” (Werezak & Stewart, 2009, p. 376 |
| Taking control |  |  |
| Explaining | “I’ve told all my good friends; and I think it is a big mistake not to tell people when you have this. Because they will wonder how come you don’t remember this? And how come you are reacting this way.” (Harris, 2012, p. 311)  “So they could understand. Because I know that it goes a bit wobbly, you know.” (Hedman et al., 2013, p. 726)  **“You can’t be blamed for falling ill … It’s better to tell how it is instead of having them talk about it behind closed doors, ‘He is a bit stupid’.” (Hellström & Torres, 2013, p. 162)**  “I’m very open about this, and often I’ll say, ‘I have Alzheimer’s’, because I have trouble ﬁnding words and things like that and I like people to know why.” (MacRae, 2008, p. 404)  **“And all of a sudden you're starting to do things that you hadn't been doing in the past. There's all sorts of things that come along with having Alzheimer's and I'd rather they know than behind my back saying, “Gee, what's wrong with him?” So that's–I look at it that way, so I let people know.” (O’Connor et al., 2018, p. 47)**  “If it’s somebody that we haven’t seen for a while then it’s, you know, I feel more at ease to say what the problem is than to ramble on not making any sense.” (Pipon-Young et al., 2013, p. 606)  “…we tell most people, because she's not just funny in the head, sort of thing. It's a medical condition rather than just being a ‐ years ago, they used to say a person carried on and a bit nutty, sort of thing.” (Stockwell-Smith et al., 2019, p. 634)  **“I just said this memory etc., isn’t working. I have seen the Consultant and the brain scan says early stages of Alzheimer’s, so if I start to stutter it is not the whisky!” (Weaks et al., 2015, p. 773)**  “I tell people ahead of time so if I make a mistake, I don’t feel silly.” (Williamson & Paslawski, 2016, p. 8) | **“Disclosing the diagnosis not only to his closest family but also to friends and a broader network was essential for Egon. He worried what others would think of him if they did not know the cause of his actions.” (Glavind, 2023, p. 5)**  “Two participants described it as important to ‘be oneself’ without trying to conceal diﬃculties.” (Hedman et al., 2013, p. 726)  **“… it became quite clear that most of the couples in this category felt that disclosure was helpful since it allowed them to avoid having others guess about their situation.” (Hellström & Torres, 2013, p. 162)**  “Others are informed in advance as a means of managing or avoiding what might otherwise be a socially awkward situation.” (MacRae, 2008, p. 404)  **“The value of disclosing as a necessary, self-protective measure for explaining unusual behavior, was raised by all members of the group.” (O’Connor et al., 2018, p. 47)** |
| Awareness and advocacy | “We have been very open about it, always. It is nothing to be ashamed of. I can’t help it. It has become much more accepted.” (Bielsten et al., 2018, p. 1725)  “We should brought dementia out of the cupboard. I tell people openly that I have it to challenge the stereotype.” (Castaño, 2020, p. 124)  “I just don’t understand why we are so ashamed. I have been told earlier on that African Americans, we will tell more people that we have breast cancer and prostate cancer than we will Alz …” (Johnson et al., 2022, p. 12)  “I think it’s very important that people [are properly informed]. It used to be that people thought of it [dementia] as being shameful, and I think of it as a sickness, like a broken arm or broken leg’.” (MacRae, 2008, p. 404)  **“It’s just really to raise awareness and to break down the stigma that’s attached to having a dementia diagnosis (…) that it’s okay to talk about it.” (Kohl et al., 2023, p. 4)**  **“… being able to talk about these things does give you more knowledge, more empowerment to be able to go out and tackle the world with the stigma. I think that's important.” (O’Connor et al., 2018, p. 49)**  **“I think it is a good idea [to tell people] … och yes, I mean you don’t hide things these days do you? … no we are not whispering about it.” (Weaks et al., 2015, p. 772)** | “In these cases, we see people with dementia documenting and sharing their experiences as a way to overcome stigma, often initially through private documentation that becomes more publicly consumed … - P8, explained that he began sharing his experiences online of living with dementia because he wanted to contribute and give back to the community …” (Johnson et al., 2022, p. 13)  **“A key motivation for participants to disclose their dementia diagnosis on social media and posting dementia-related information was to raise awareness about the condition. They felt that dementia was not well understood by society and wanted to combat the negative assumptions they felt many people without dementia had about the condition.” (Kohl et al., 2023, p. 4)**  “Generally there was a concern that others with a limited understanding of dementia would react to the participants by prejudging and stereotyping them and this would threaten their unique individual identity. Some participants were therefore keen for others to be better informed about the condition.” (Langdon et al., 2007, p. 995)  “Stating ‘I ﬁnd no shame in it’, Malcolm believed people should know more about AD and told ‘everybody every chance’ he got. Ed, also believing that AD was ‘nothing to be ashamed of’, told ‘anyone that [wanted] to listen’.” (MacRae, 2008, p. 400)  “Emma thought that being open about dementia might strengthen her current relationships by raising awareness of dementia amongst others.” (Thoft & Ward, 2022, p. 1730)  “Study participants saw openness as a way of decreasing embarrassment and increasing understanding of dementia, particularly in public situations.” (Williamson & Paslawski, 2016, p. 8)  “’Openness about the diagnosis’ was perceived to lead to less personal embarrassment and increase public awareness and understanding.” (Windle et al., 2023, p. 2362) |
| Reduction of stress and burden | The simple act of letting [the family] know that you have Alzheimer's reduces tensions, problems () It's much better to disclose and let them know [about the diagnosis]. (Gajardo et al., 2021, p. 189; translated from Spanish)  “I will not be ashamed of having Alzheimer’s (). I will tell it to all that I get in touch with. () Then I don’t have to make myself weird, or be aﬀected, because I could not stand that.” (Hedman et al., 2013, p. 726)  **“And you hope by putting on [to Facebook] something that has happened to you, that you help somebody else, that somebody else is able to say, “Oh, that happened to me and that worked for them or whatever”, you know?” (Kohl et al., 2023, p. 6)**  **“I ﬁnd this very helpful and I put this [badge] on my chest because, as you said, ‘You don't look like you are a disabled person.’ So what I do is, when I go to the banks, because when I talk to strangers I get stressed and I cannot express myself. So with this, automatic when I'm having the problem–I just go–and they seem to be very understanding–clerks, cashiers, and stuﬀ.” (O’Connor et al., 2018, p. 47)**  “But there are many others with diﬀerent hobbies, such as [NAME] who writes poems and brain games, and we need to hear from them so that we can help others who are starting on the dementia journey.” (Talbot & Coulson, 2023, p. 4)  **“It’s more stressful than it should be and ... I think where the stress of it has come is trying to keep everything on the surface going while things are just not really quite the same.” (Weaks et al., 2015, p. 773)** | “Informing others about having dementia was natural and empowering for couples. Informing others contributed to reduced stress due to not having to cover up symptoms and contributed to conditions for other people to be supportive and understanding.” (Bielsten et al., 2018, p. 1725)  **“For this group, sharing the diagnosis was a way to cope and ensure that everyone was in the loop and prepared to offer support, such as Egon’s call for future support for his family.” (Glavind, 2023, p. 6)**  **“Through posting content on social media, interviewees described assuring other people with dementia that not every day was the same and positive things were part of living with dementia too.” (Kohl et al., 2023, p. 6)**  “By sharing these experiences of ‘living’ with dementia, some users hoped that they could help individuals who had recently been diagnosed.” (Talbot & Coulson, 2023, p. 4)  “They were open and honest about their situation, finding it helped ease the stress of trying to hide the dementia and made it easier to get help.” (Thoft & Ward, 2022, p. 1730)  “Several participants with dementia noted that by sharing the news of their diagnosis with others, there was a wider range of support that was available should they need it … By sharing his diagnosis with others, he stated that his care partner was able to advocate for him more readily and increase support for both of them.” (Williamson & Paslawski, 2016, p. 8)  “’Openness about the diagnosis’ was perceived to lead to less personal embarrassment and increase public awareness and understanding.” (Windle et al., 2023, p. 2362) |

*Note*. Bold quotes indicate studies whose primary research question focused on self-disclosure.
